# Discrimination of Entamoeba Spp. in children with dysentery

**Published:** 2014

**Authors:** Mitra Sharbatkhori, Ehsan Nazemalhosseini-Mojarad, Fatemeh Cheraghali, Fatemeh Soghra Maghsoodloorad, Heshmatolla Taherkhani, Mohammadali Vakili

**Affiliations:** 1 Infectious Diseases Research Center, Golestan University of Medical Sciences, Gorgan, Iran; 2 Department of Parasitology and Mycology, School of Medicine, Golestan University of Medical Sciences, Gorgan, Iran; 3 Gastroenterology and Liver Disease Research center, Shahid Beheshti University of Medical Sciences, Tehran, Iran; 4 Department of Pediatric Diseases, Golestan University of Medical Sciences, Gorgan, Iran; 5 Department of Community Medicine, School of Medicine, Golestan University of Medical Sciences, Gorgan, Iran

**Keywords:** Entamoeba histolytica, Entamoeba dispar, children, dysentery, PCR, Gorgan, Iran

## Abstract

**Aim**: The present study was performed in order to differentiate *E. histolytica *and *E. dispar *in children from Gorgan city, using a PCR method.

**Background**: Differential detection of two morphologically indistinguishable protozoan parasites *Entamoeba histolytica *and *E. dispar *has a great clinical and epidemiological importance because of potential invasive pathogenic *E. histolytica *and non-invasive parasite *E. dispar*.

**Patients and methods**: One hundred and five dysentery samples were collected from children hospitalized in Taleghani hospital in Gorgan city. The fecal specimens were examined by light microscopy (10X then 40X) to distinguish *Entamoeba *complex. A single round PCR amplifying partial small-subunit rRNA gene was performed on positive microscopy samples to differentiate *E. histolytica/ E. dispar* *and* *E*. *moshkovskii *from each other.

**Results**: Twenty-five specimens (23.8%) were positive for *Enramoeba *complex in direct microscopic examination. PCR using positive controls indicated *E. histolytica and E. dispar* in two (2/25, 8%) and three (3/25, 12%) samples, respectively.

**Conclusion**: There is a warrant to performing molecular diagnosis for stool examination at least in hospitalized children in order to prevent incorrect reports from laboratories and consequently mistreating by physicians.

## Introduction

Amoebiasis is still mentioned as one of the main health problems in tropical and subtropical regions (1). The true prevalence of infection caused by *Entamoeba histolytica* is unknown for most areas of the world (2). 

*E. histolytica* causes widespread mortality and morbidity worldwide through diarrheal disease and abscess establishment in parenchymal tissues such as liver, lung, and brain (3). In contrast, other amoebae that infect humans include *E. dispar*, *E. moshkovskii*, *E. coli*, *E. hartmanni*, and *Endolimax nana*, have been considered nonpathogenic (4).

*E*. *histolytica*, *E*. *dispar*, and *E*. *moshkovskii *are morphologically indistinguishable but are different biochemically and genetically. Although *E*. *histolytica *is recognized as a pathogen, the ability of the other two species to cause disease is unclear (5). It is also worthy to note that until recently the differentiation of *E. histolytica* from the non-pathogenic amebic species was not possible (6, 7).

The epidemiology of *E. histolytica* in Iran is poorly understood. Fortunately, several studies over the last decade have begun to evaluate the prevalence or incidence of *E. histolytica* in specific populations of Iran, these studies were utilized different molecular methods in order to differentiate the non-pathogenic *E*. *dispar* or *E*. *moshkovskii *from the pathogenic *E. histolytica* (8-16). In this study, we used the well-characterized diagnostic tests (single round PCR) to examine the prevalence of *E. histolytica,* *E. dispar* and E. *moshkovskii *infections in children with dysentery in Golestan province, Iran.

## Patients and Methods

From January 2010 to September 2012, 105 dysentery samples were collected from children hospitalized in Taleghani hospital in Gorgan city, the capital of Golestan Province, located in northern Iran and south east of Caspian sea. Socio-demographic and clinical data were collected from the child’s parents and medical records.

Stool specimens were screened microscopically using direct slide smear for the presence of *Entamoeba* spp. (13). Genomic DNA was extracted directly from stool specimens were microscopically positive by using a QIAamp® DNA Stool Kit (QIAGEN) according to the manufacturer's instructions. The extracted DNA was stored at -20°C until PCR amplification. A single–round PCR reaction and primers sets amplifying partial small-subunit rRNA gene were used as described previously (13, 17, 18). The sequence of the forward primer used was conserved in all three *Entamoeba *spp., but the reverse primers were specific for apiece. The expected PCR products from *E. histolytica, E. dispar *and *E. moshkovskii *were 166 bp, 752 bp, and 580 bp, respectively (17).

Amplification of each species–specific DNA fragment started with an initial denaturation at 94°C for 3 min, followed by 30 cycles of 94°C for 1 min, 58°C for 1 min, and 72°C for 1 min, with a final extension at 72°C for 7 min (14). PCR products were visualized with ethidium bromide staining after electrophoresis on 1.5% agarose gels. DNA isolated from axenically grown *E.* *histolytica KU2*, *E. dispar AS 16 IR *and *E. moshkovskii Laredo *(ATCC accession no. 300 42) (13) were used as positive controls. Medical Research Ethics Committee of Golestan University of Medical Sciences approved the study.

**Figure 1:**
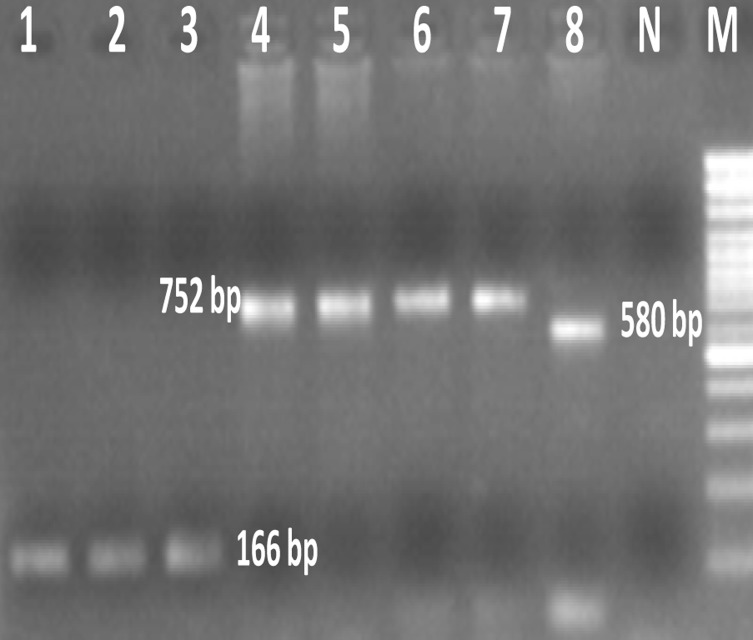
Agarose gel electrophoresis of *Entamoeba* species using single–round PCR. Lanes 2-3: *E. histolytica* – positive isolates; Lane 5-7: *E. dispar *– positive isolates; Lanes 1, 4 and 8: *E. histolytica* , *E. dispar and E. moshkovskii* positive controls, respectively. N: Negative control. M: 100 bp ladder DNA size marker

## Results

Out of the 105 children with dysentery, 25 (23.8%) cases were infected with *E. histolytica/E. dispar/E. moshkovskii *complex. After DNA extraction, the single-round PCR was carried out to differentiate the *Entamoeba *spp. of 25 samples that were microscopically positive. Three (12%) *E. dispar* and 2 (8%) *E. histolytica *were detected by PCR (Figure 1). Infection of *E. moshkovskii *was not observed in this study. 

Amplification produced fragments of 166bp, 752bp and 580bp corresponding to the expected products from *E. histolytica*, *E. dispar and E. moshkovskii*, respectively.

## Discussion

Infection with *E. histolytica *is a severe health problem in many tropical and subtropical areas of the world, especially in developing countries such as Iran (19, 20). 

It is now known that most of human cases of infection with *E. histolytica/E. dispar *are actually *E. dispar*. *E. dispar *is non-pathogenic, and requires no treatment. Because of this, differential diagnosis of the pathogen *E. histolytica *from the commensally *E. dispar *is of the utmost importance (21).

Most epidemiological studies for *E. histolytica *infection were performed before the acceptance of *E. histolytica/E. dispar* and* E. moshkovskii* as distinct species. There is a clear need to perform new epidemiological studies to distinguish these three species of *Entamoeba *and to find true prevalence of *E. histolytica *species (14).

The results of this study clearly show that, microscopy is not a sensitive and reliable technique for diagnosing amebic dysentery as well as differentiation of *E. histolytica *from *E. dispar* and *E. moshkovskii*, because most amebic infections in this population were due to other nonpathogenic *Entamoeba species* (17). In a previous study in Gonbad city 16 (69.5%) from 23 microscopic positive isolates for *Entamoeba* species were amplified in PCR as *E. dispar*, none of the isolates (0%) revealed as *E. histolytica* and the seven isolates were not amplified in PCR. The higher prevalence of *E. dispar* is in concordance with our study (10). The 20 negative PCR isolates (30.43%) with both two sets of *E. histolytica* and *E. dispar *primers in this study might be due to lack of enough DNA template, PCR error or misdiagnosis with some other *Entamoeba* species. However, this conjecture should be confirmed by the further development of molecular diagnosis for other nonpathogenic *Entamoeba species* commonly found in humans, such as *E. coli *and *E. hartmanni *(17).

While the isolates were achieved typically from patients who suffered from dysentery, we found no *E. histolytica *among the isolates and observed no correlation between the presences of *Entamoeba *spp. and clinical symptoms, such as abdominal pain, diarrhea or nausea. It may be that the clinical symptoms of some patients were due to viral or bacterial pathogens not detected by the tests that had been run as in a valid study, Kermani et al. showed that Enteroaggregative *Escherichia coli *was the most prevalent pathogen in both persistent and acute diarrhea children admitted to a pediatric hospital (22).

In conclusion, the finding of our study emphasis to perform molecular diagnosis for stool examination at least in hospitalized children in order to prevent incorrect results from medical laboratories and consequently mistreating by physicians.

## Acknowledgment

The authors would like to thank Fatemeh Malek, Zeinab Golalikhani, Hamid Mirkarimi and Akbar Mirbazel in the Taleghani hospital who helped to collect the samples. This work was financially supported by Golestan University of Medical sciences, project code: 8912240184.
